# Advanced Metallized Nanofibers for Biomedical Applications

**DOI:** 10.1002/advs.202302044

**Published:** 2023-08-02

**Authors:** Wei Sang, Ruiping Zhang, Xiangyang Shi, Yunlu Dai

**Affiliations:** ^1^ Cancer Center and Institute of Translational Medicine Faculty of Health Sciences University of Macau Macau SAR 999078 China; ^2^ Institute of Medical Technology Shanxi Medical University Taiyuan 030001 China; ^3^ The Radiology Department of First Hospital of Shanxi Medical University Taiyuan 030001 China; ^4^ State Key Laboratory for Modification of Chemical Fibers and Polymer Materials Donghua University Shanghai 201620 China; ^5^ MoE Frontiers Science Center for Precision Oncology University of Macau Macau SAR 999078 China

**Keywords:** biomedicine, biosensor, drug delivery, metallized nanofibers, wound healing

## Abstract

Nanofibers are long, wire‐like materials with nanoscale diameters and specific length diameter ratios. Nanofibers have porous reticular networks with remarkably high specific surface areas and significant interconnectivity between pores, allowing for the chemical modification and loading of drugs. Metallized nanofibers are novel materials that enhance the performance of attributes of conventional nanofibers by combining metals with nanofibers through electrostatic spinning doping, chemical modification, and loading approaches. Due to their unique physical and chemical properties, metallized nanofibers are diverse, rapidly developed materials in the fields of physical chemistry, materials science, and battery preparation. To date, with improvement in advanced preparation techniques and biocompatibility levels for materials, metallized nanofiber applications are gradually expanding into the biomedical field due to their excellent thermal and electrical conductivities and unique metal properties. In this review, the applications of metallized nanofibers in biomedicine are summarized. It is suggested to prepare metallized multifunctional nanofibers for tissue engineering, drug delivery, tumor treatment, wound healing, and biosensing applications by taking safety and stability as the main material selection guidelines. Finally, the development of nanofibers for biomedical applications is summarized and discussed.

## Graphical abstract

Nanofibers are nanomaterials with remarkable high surface properties. With diverse modifications, nanofibers possess optical, thermal, electrical, and magnetic properties, which can be widely applied in high precision fields. In this review, nanofibers based on metallic materials for biomedical applications are summarized for the first time. This review will allow researchers to better understand diverse applications of metallized nanofibers in biomedicine.

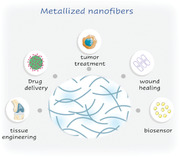

## 1 Introduction

Natural fibers, such as cotton, hemp, and silk were used by humans as early as the Neolithic era to cover the body and keep warm. With the development of the chemical and polymer material industries, a series of synthetic polymer fibers have been designed and synthesized; these fibers are not limited to clothing and home textiles, instead expanding to applications in the environment, energy, medicine, and other high precision technologies. In 1990, the first International Conference on Nanoscience and Technology was held to mark the official establishment of the field of nanotechnology. The rise in nanotechnology has led to the vigorous development and widespread application of nanomaterials, making nanomaterials an important force in the progress of science and technology. Nanofibers are one‐dimensional nanomaterials with remarkably high surface properties. With diverse modifications, nanofibers possess optical, thermal, electrical, and magnetic properties, which can be widely applied in high‐precision fields, such as aerospace, national defense, environmental management, electronic energy, and biomedicine.

Metallized nanofibers, which are new types of nanomaterial, have wide ranges of promising applications in the biomedical field. This vast applicability arises because metal ions have many unique physicochemical properties, such as large specific surface areas and good surface, magnetic, optical and electronic properties, providing them many unconventional uses in biomedicine. First, metal particles have large specific surface areas, making them very versatile for biomedical applications. For example, metal particles can be used in biosensors to detect biomolecules by detecting chemical reactions on the surfaces of metal particles. In addition, metal particles can be used for drug delivery, where the solubility and bioavailability of a drug can be increased by wrapping the drug on the surfaces of metal particles, thus improving its efficacy. Second, the surface properties of metal particles can be modulated by surface modification, thereby enhancing their applicability in biomedical fields. For example, surface modification of metal particles can improve their biocompatibility, enhance their stability, and change their surface charge. In addition, surface modification of metal particles can be used to prepare nanomaterials with specific functions, such as targeted drug delivery systems and bioimaging probes. Third, some metal particles have magnetic properties, making them very widely used in biomedical fields. For example, magnetic metal particles can be used in magnetic resonance imaging, where the magnetic properties of metal particles are controlled to image biological tissues. In addition, magnetic metal particles can be used for magnetic targeted drug delivery, thus improving the efficacy of drugs by guiding them to specific targets through magnetism. Fourth, some metal particles exhibit special optical properties, such as surface plasmon resonance and fluorescence, making them very versatile in biomedical applications. For example, metal particles can be used for cell imaging by controlling the optical properties of metal particles to image cells. In addition, metal particles can be used in biosensors to detect biomolecules by detecting the fluorescence signals on the surfaces of metal particles. Finally, some metal particles exhibit special electronic properties, such as the presence of quantum dots and electrical conductivity, making them very versatile in biomedical applications. For example, metal particles can be used for fluorescence imaging, in which biomolecule imaging is achieved by controlling the electronic properties of metal particles. In addition, metal particles can be used for electron transport by controlling the electrical conductivities of metal particles. Metal particles, as new nanomaterials, have many application prospects in the biomedical field. The physicochemical properties of metal particles allow them to be used in various biomedical applications, such as drug delivery, cell imaging and biosensing. These applications should lead to innovation and development in the biomedical field.

Much research progress in the field of nanomedicine has been devoted to nanofibers modified with metals. Various metal properties, such as ductility, thermal and electrical conductivity, dimensional stability, and high formation, can be used in numerous nanofiber applications. For example, metallized nanofibers exhibit excellent plasticity and antibacterial properties, and they can be utilized as nanofiber materials in the field of tissue engineering. In addition, modifying specific drugs on their nanofibers can be beneficial for local targeting and localization therapy. Another application of metallized nanofibers is drug delivery. The interactions between metals and materials can form a porous network structure; filling the pores with drugs can achieve localized and targeted release, thus improving drug efficiency while reducing the consequences of drugs on main tissues and organs. Due to the specific conductivities of metals, a series of sensors can be prepared to determine the contents of biomolecules. Therefore, metallized nanofibers are versatile and have far‐reaching implications in biomedicine.

**Figure 1:**
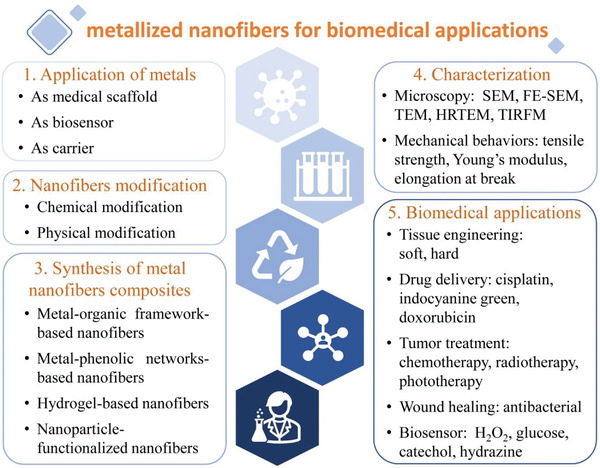
Schematic illustration showing metallized nanofibers for design, modification, characterization, and their biomedical applications.

In this review, nanofibers based on metallic materials for biomedical applications are summarized for the first time. In general, the overall review is organized into five sections (**Figure** **1**). The first part involves a description of the reasons for incorporating metal elements when preparing nanofibers, and the characteristics and applications of metals. The second part features a list of the diverse modification methods and characteristics of nanofibers. Next, different preparation methods and morphological characteristics of nanofibers are presented. The fourth part covers the characterization of metallized nanofibers. The last section focuses on outlining the biomedical applications of metallized nanofibers. Overall, this review allows researchers to better understand the diverse applications of metallized nanofibers in biomedicine for solving clinical medical challenges.

## 2 Applications of Metals

**Figure 2:**
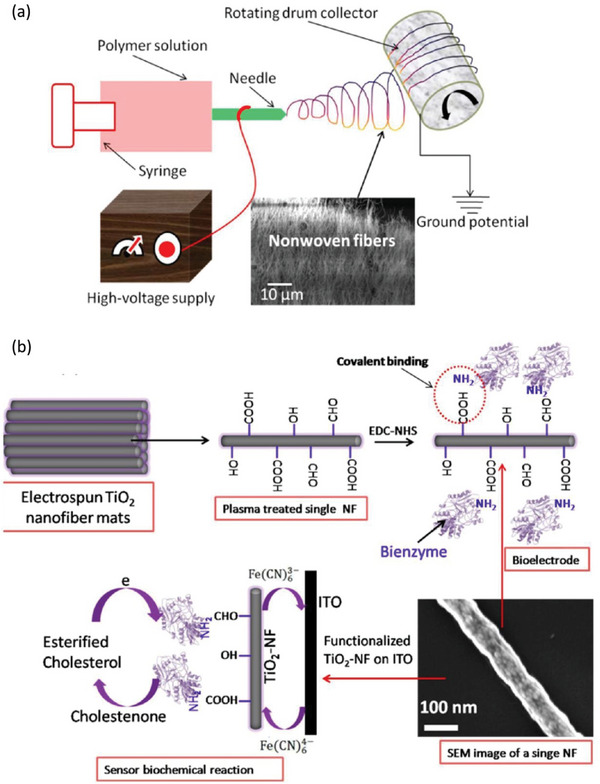
a) Schematic illustration showing the electrospinning settings of synthetic TiO_2_ nanofibers. b) The principle of mesoporous TiO_2_‐NF for esterified cholesterol detection. Reproduced with permission.^[^ ^2^ ^]^ Copyright 2014, American Chemical Society.

To date, innovative multifunctional materials with pharmaceutical and biomedical applications are extensively available for nanofiber fabrication, improving the unique properties of nanofibers such as their biocompatibility and biodegradability, which have attracted the attention of interdisciplinary researchers. Combining metals and nanofibers is an approach that has been developed in recent years. Among numerous metal nanoparticles, silver nanoparticles occupy an extremely valuable position in the field of microelectronics due to their excellent electrical conductivity. In addition, the surface and quantum size effects of silver nanoparticles have special application potential regarding surface‐enhanced Raman scattering and medical applications. Furutani et al. modified the protocol for the precipitation and immobilization of silver nanoparticles on cellulose nanofibers; this protocol involves exploiting the thermal energy generated by different processing conditions of a high‐pressure wet jet.^[^ ^1^ ^]^ Other materials have demonstrated potential applications in nanomedicine. Mondal et al. prepared biofunctionalized mesoporous titanium dioxide nanofibers for the relatively rapid and sensitive electrochemical detection of esterified cholesterol.^[^ ^2^ ^]^ In this work, the authors enabled the detection of cholesterol by covalently modifying cholesterol esterase and cholesterol oxidase biomolecules on titanium dioxide nanofibers (**Figure** **2**). The enzyme‐functionalized titanium dioxide nanofibers‐based biosensor exhibits a high signal‐to‐noise ratio and excellent voltammetric and catalytic performance characteristics; these attributes are present with improved detection limits and sensitivities, and they are highly reproducible. Figure 2a,b shows a stainless steel rotating drum collector wrapped with aluminum foil, which is used as a collector for electrospun fibers; its potential biomolecule detection is a promising platform for the development of miniaturized devices for biosensing applications. Xue et al. prepared an oxygen sensor from polycarbonate‐polycaprolactone core–shell polymer nanofibers to detect oxygen depletion at tumor sites.^[^ ^3^ ^]^ The monolithic nanofibers are composed of transition metal porphyrin luminescent probe complexes embedded in transparent, highly stable, and breathable polycarbonate cores. Experimental results show that the nanofibers have high sensitivity and fast response performance characteristics and that the biosensor can localize and image hypoxic regions around glioblastoma cell aggregates.

Therefore, multifunctional nanofibers can be prepared based on the properties of many metals. Section six presents a summary of some other functions of metallized nanofibers.

## 3 Synthesis and Modification of Nanofibers

Nanofibers are conventionally synthesized by electrospinning technology.^[^ ^4^ ^]^ The conventional electrostatic spinning device is mainly composed of four parts: a capillary tube, collection device, precision injection pump, and high voltage power supply. At the beginning of electrostatic spinning, the polymer fluid is stored in the capillary by surface tension. When the applied electric field acts on the top of the capillary, the fluid surface generates a large amount of electrostatic charge. The surface tension of the droplet at the top of the capillary is weakened by electrostatic repulsion and is gradually stretched to form a charged cone. When the electric field strength increases to a critical value, the charged fluid is ejected from the apex to form a charged jet. The jet is first stretched into a straight line by a certain distance and then bent in a circular or spiral manner. Finally, either the solvent evaporates and condenses or the melt cools and solidifies to form polymer fibers, which are deposited on a collection plate by the electric field force. In addition to classical electrospinning techniques, researchers have explored several variants, including multineedle, needleless, coelectrostatic spinning, and coaxial electrospinning methods. These approaches have various advantages, and all enable the synthesis of metallized nanofibers.^[^ ^5^ ^]^

Progress has been made in fabrication technology for metallic fibers and metallic hybrid nanofibers. The synthesis of metallized nanofibers can be achieved by a combination of electrospinning and metallization.^[^ ^6^ ^]^ A relatively high amount of research is aimed at doping metal nanoparticles into electrospun hybrid nanofibers, thus stochastically distributing nanoparticles in nanofibers. This approach unevenly distributes metals and features uncontrollable processes.^[^ ^7^ ^]^ Other widely studied strategies include in situ composites combined with electrospinning approaches and in situ reductions in metal salts combined with the growth and synthesis of metals on nanofiber surfaces.^[^ ^8^ ^]^

Nanofiber modification includes chemical modification and physical modification. Chemical modification refers to changing the chemical structure of nanofibers through chemical reactions to achieve specific properties or functions; physical modification refers to changing the morphologies, structures or properties of nanofibers through physical techniques to achieve specific properties or functions.

Metal ion‐doped bioactive glass nanofibers have emerged as promising materials for tissue regeneration applications due to their unique properties and capabilities. These nanofibers are composed of glass doped with metal ions, allowing them to interact with biological tissues and promote regeneration. The metal ions added to the bioactive glass nanofibers include calcium, magnesium, and zinc, which significantly affect tissue regeneration. Ca^2+^ is essential for forming bones, teeth, and cartilage, while Mg^2+^ plays vital roles in muscle and nerve function. Zn^2+^ is important for tissue regeneration because it is involved in collagen formation and wound healing. Metal ion‐doped bioactive glass nanofibers are produced using electrospinning techniques, thus producing fibers with diameters in the nanometer range. The fibers are coated with a layer of bioactive glass, enhancing their biocompatibility and bioactivity. The bioactive glass layer provides a surface for the metal ions to interact with the surrounding tissues, promoting regeneration. A key advantage of metal ion‐doped bioactive glass nanofibers is their ability to mimic the structure and composition of a natural extracellular matrix (ECM). The ECM is a complex network of proteins and other molecules that provide structural support to cells and tissues. By mimicking the ECM, bioactive glass nanofibers can provide a scaffold for cells to grow and regenerate, thus forming new tissues. Metal ion‐doped bioactive glass nanofibers have many applications in tissue regeneration. Among their applications, these nanofibers can promote the regeneration of bone, cartilage, and nerve tissues. In bone regeneration, bioactive glass nanofibers have been shown to enhance the formation of new bone tissue, thus improving leading to improved healing of fractures and other bone injuries. In cartilage regeneration, the nanofibers can promote the growth of new cartilage tissue; this tissue can then be used to repair damaged joints. In nerve regeneration, nanofibers promote the growth of new nerve tissue, which can be used to repair damaged nerves. Metal‐glass nanofibers can mimic the structure and composition of a natural ECM, providing a scaffold for cells to grow and regenerate. Metal ions added to bioactive glass nanofibers significantly affect tissue regeneration, making them a versatile material for numerous applications. With further research and development, metal ion‐doped bioactive glass nanofibers can revolutionize the field of tissue regeneration and improve the lives of millions of people worldwide.

## 4 Morphological Classification of Metallized Nanofiber Composites

The application of metallized nanofibers is an emerging research topic in biomedicine. Researchers have designed and synthesized nanofibers and nanomaterials with different morphologies for highly specialized applications. Different functional nanofibers have been established according to their different morphologies, including metal‐organic frameworks (MOFs) and metal‐phenolic networks (MPNs) combined with nanofibers, nanofibers forming different hydrogel analogs, and nanofibers combined with nanoparticles.^[^ ^9^ ^]^ The multitude of morphologies has diversified the compositions and expanded the applications of nanofibers.

### 4.1 MOFs‐Based Nanofibers

**Figure 3:**
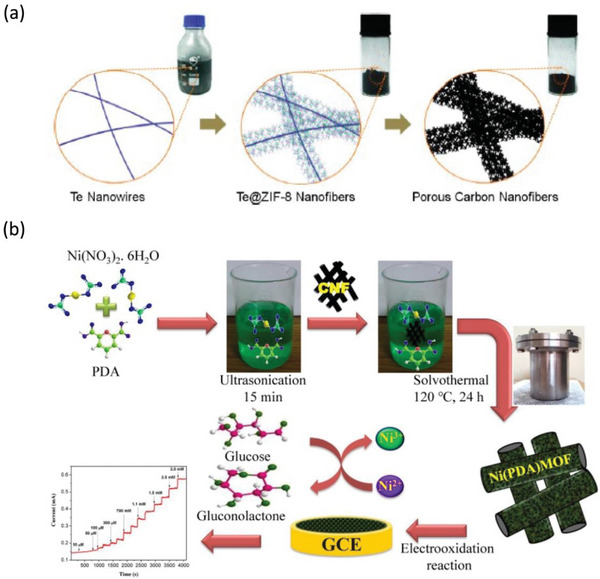
a) Synthesis method of porous carbon nanofibers. Reproduced with permission.^[^ ^16^ ^]^ Copyright 2014, American Chemical Society. b) Schematic diagram of the preparation method of Ni(PDA)MOF@CNF pad and the principle of the pad for glucose detection. Reproduced with permission.^[^ ^12^ ^]^ Copyright 2022, Elsevier.

MOFs are a class of organic materials with adjustable pore sizes formed by the self‐assembly of organic ligands and metal centers. Over 20 000 MOFs have been synthesized by selecting metal centers and organic ligands, which have great potential in many fields. Furthermore, other binding forces are present in MOFs, which play important roles in the structures of MOFs, such as hydrogen bonding, van der Waals forces, and *π*–*π* stacking. Because of these advantages, MOFs have been widely used in many applications, including gas adsorption and separation, sensors, drug mitigation, and catalytic reactions.^[^ ^10^ ^]^ The combination of MOF structures with nanofibers is useful for corresponding specialties, providing complementary advantages. Zhang et al. reported a new strategy for synthesizing one‐dimensional ZIF‐8 nanofibers using tellurium nanowires as a template, which can be transformed into doped carbon nanofibers by calcination (**Figure** **3**a).^[^ ^11^ ^]^ These co‐doped carbon nanofibers exhibit excellent electrocatalytic activities and they are highly tolerant to diverse applications for long time periods. To obtain Te@ZIF‐8, hydrothermal conditions are initially employed to acquire ultrathin tellurium nanowires (TeNWs). Subsequently, zinc nitrate and 2‐methylimidazole are mixed in a methanol solution of TeNWs at room temperature. To preserve the 3D scaffold structure of ZIF‐8 nanofibers on a microscale, a freeze‐drying technique is utilized. The final step involves transferring the dried ZIF‐8 nanofiber powder to a temperature‐programmed furnace in the presence of nitrogen gas flow. Heat treatment is provided at 200 °C for 6 h, followed by pyrolysis at 1000 °C for 8 h, thus forming highly porous doped carbon nanofibers. Because the derived porous carbon and metal oxide materials are resistant to methanol crossover effects, they can be applied to various applications in physical chemistry and bioengineering. Another study involved a focus on the preparation of MOF‐based nanofibers for nonenzymatic glucose detection. Dey et al. reported Ni(PDA)MOF@CNF, which is an in situ self‐assembly of Ni‐MOFs loaded on carbon nanofiber mats (Figure 3b).^[^ ^12^ ^]^ The porous structure and large specific surface area of the MOF can bind well to the detected biochemicals, greatly improving the detection sensitivity. This sensor can maintain accurate detection data under various physiological conditions, and it is very useful for monitoring glucose and human sweat lactate.

### 4.2 MPN‐Based Nanofibers

Compared with MOFs, MPN materials have relatively soft structures. Metal ions and polyphenols can easily and rapidly self‐assemble into MPNs. MPNs have simple and green assembly preparation processes; additionally, they can be loaded and adhered to various entities. Therefore, MPNs have the great potential for applications in the fields of bionics, materials science, and biomedicine.^[^ ^13^ ^]^ Electrospun ultrafine nanofibers, due to their unique structures, can mimic the extracellular matrix state and promote vascular cell interactions and functional activity. However, the poor adhesion of some synthetic polymers can lead to the defective cell formation of blood vessels. By exploiting the adhesive properties on the surfaces of MPNs, Li et al. assembled tannic acid and Fe^3+^ ions in coordination on polycaprolactone (PCL) nanofibers to form TA‐Fe^3+^‐coated PCL nanofibers.^[^ ^14^ ^]^ MPN‐coated PCL nanofibers facilitate cell growth and tissue repair through the stable adhesion, free radical scavenging and ion‐releasing properties of MPNs. The MPN‐based nanofiber fabrication process is mature, simple, and fast, facilitating biomedical and clinical applications. First, MPN‐based nanofibers have excellent biocompatibility and biodegradability, making them promising for numerous biomedical applications. Second, MPN‐based nanofibers can modulate cell behavior by controlling the diameters and morphologies of the fibers, which is of great importance for certain fields, such as cell culture and tissue engineering. In addition, MPN‐based nanofibers can achieve selective adsorption and release of biomolecules by modulating the functionalized groups on the fiber surface, which has potential applications in the fields of drug delivery and biosensing. Finally, MPN‐based nanofibers can be used with other biomaterials (e.g., bioceramics, biopolymers, etc.) for many biomedical applications.

### 4.3 Hydrogel‐Based Nanofibers

**Figure 4:**
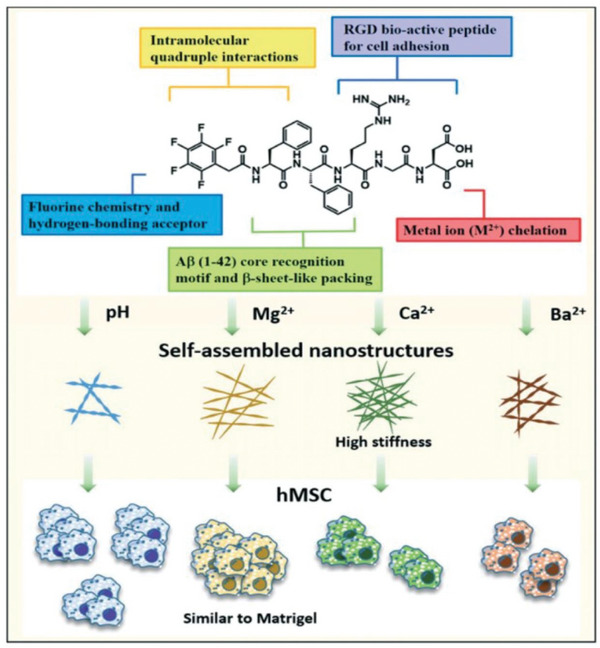
The self‐assembly of a pentapeptide with bioactive PFB end‐capping leads to the formation of supramolecular nanostructures with different cross‐linking densities, and the matrix‐dependent multicellular assembly of hMSCs in a three‐dimensional hydrogel culture. Reproduced with permission.^[^ ^16^ ^]^ Copyright 2022, Royal Society of Chemistry.

The hydrogel‐based nanofiber morphology enables its utilization for targeted applications. The advantages of hydrogel nanofibers include high water content, excellent biocompatibility, and low density. Hydrogel nanofibers are composed of hydrophilic polymers that can absorb and retain large amounts of water, making them highly suitable for several applications, such as drug delivery, tissue engineering, wound healing, and biosensing. A key advantage of hydrogel nanofibers is their high surface area‐to‐volume ratios, allowing for the efficient delivery of drugs and growth factors to targeted tissues. Additionally, their high porosities and interconnected pore structures facilitate the exchange of nutrients and waste products, thus promoting cell growth and tissue regeneration. Hydrogel nanofibers possess excellent mechanical properties, enabling them to mimic the natural extracellular matrix of tissues and provide structural support to cells. Furthermore, these nanofibers can be easily fabricated into various shapes and sizes, allowing for customization and versatility in their use. Overall, the advantages of hydrogel nanofibers make them a promising material for several biomedical applications, and their potential for further development and optimization is significant. Thus, the development of hydrogel nanofibers is of significance for multiple biomedical application, including drug delivery, tissue engineering, wound dressing, and photoluminescence.^[^ ^15^ ^]^ Mohiuddin Mohammed et al. fabricated a hydrogel‐based biodegradable material with tunable mechanical stiffness and biological functions (**Figure** **4**).^[^ ^16^ ^]^ In this study, the authors demonstrate the self‐assembly of the pentapeptide Phe‐Phe‐Arg‐GlyAsp (FFRGD) with a fluorinated benzyl group at its N‐terminal end, forming stable supramolecular hydrogels with a twisted nanoribbon morphology at a physiological pH level. The addition of Mg^2+^ stimulates the formation of hydrogels with twisted nanofibrous networks and enhanced mechanical properties, such as a storage modulus of 3.6 kPa. The hydrogel triggered by Ca^2+^ proceeds through strong metal ligand chelation with a high‐crosslink density nanofiber morphology, showing an increased storage modulus of 47 kPa. However, with Ba^2+^, the hydrogel shows weak mechanical properties with a gel modulus of 0.69 kPa due to the poor cross‐linking of metal ligands. The obtained hydrogels exhibit a loosely cross‐linked twisted nanoribbon morphology. All the hydrogels show good biocompatibility with two different cell lines, namely, human mesenchymal stem cells (3A6‐RFP) and mouse fibroblasts (L929).

Alloyed nanofibers can better utilize multiple metals rather than having only one metal function, such as enhancing the stability and activity levels of nanofibers. Zhai et al. reported a study on nanoalloys doped with copper and silver in electrospun hydrogel nanofibers.^[^ ^17^ ^]^ This study involved a simple, controllable, and versatile process. Copper‐silver bimetallic hydrogel nanofibers show good selectivity for reducing 4‐nitrophenol and high catalytic activity for reducing methylene blue. Hydrogel nanofibers can be used as biomimetic materials for various functions, such as enzyme catalysis and probe detection.

### 4.4 Nanoparticle‐Functionalized Nanofibers

**Figure 5:**
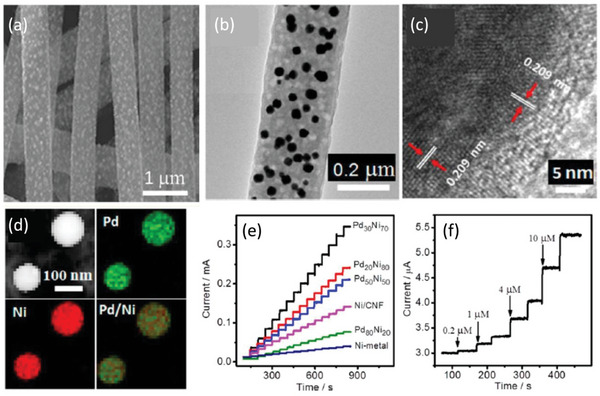
a) The SEM images of Pd_30_Ni_70_/CNF nanocomposites (Tc = 850 °C). b) The TEM images of Pd_30_Ni_70_/CNF prepared at 900 °C. c) The HRTEM images of Pd_30_Ni_70_/CNF prepared at 900 °C. d) The HAADF‐STEM images of Pd_30_Ni_70_/CNF prepared at 900 °C. e) Experimental results of Ni‐metal, NiCFP, and Pd‐NiCFP electrodes based on different Pd/Ni/CNF ratios upon continuous addition of 0.2 mm glucose. f) Current response of the Pd_30_Ni_70_CFP electrode when different concentrations of glucose solution were continuously applied. Reproduced with permission.^[^ ^20^ ^]^ Copyright 2014, American Chemical Society.

In recent years, researchers have explored numerous approaches for modifying the surface properties and improving the mechanical strengths of polymeric nanofibers to enhance the adsorption capacity.^[^ ^18^ ^]^ An excellent method includes the electrostatic spinning of nanoparticles dispersed in polymer solutions into polymer nanofibers. The encapsulated nanoparticles should be well‐dispersed in the polymer solution and exhibit the ability to separate from the surfaces of nanofibers. Lamas‐Ardisana et al. designed and prepared LOx/PtNp‐CNF‐PDDA/SPCEs as disposable biosensors for lactate oxidase.^[^ ^19^ ^]^ The authors prepared the nanofibers in the following manner, which is described briefly. Platinum nanoparticles are chosen to decorate the carbon nanofibers, and the surfaces are modified by dispersion in the poly(diallyldimethylammonium) chloride solution. The obtained sensors are sensitive and cost controllable. By comparing commercial sensors with LOx/PtNp‐CNF‐PDDA/SPCEs for changes in lactate before and after exercise, the material is found to have comparable advantages to commercial options; therefore, the researchers believe that this sensor has far‐reaching implications for biomaterial applications. In addition, Guo et al. prepared Pd‐Ni alloy nanoparticle/carbon nanofibers for sugar analysis (**Figure** **5**).^[^ ^20^ ^]^ Briefly, the nanofibers are formed by the electrostatic spinning of the precursor polyacrylonitrile/Pd(acac)_2_/Ni(acac)_2_ by heat processing. Figure 5a shows typical scanning electron microscopy (SEM) images of PdNi/CNF with Pd/Ni molar feed ratios. Figure 5b shows typical transmission electron microscopy (TEM) images of PdNi/carbon nanofibers. Figure 5c shows typical high resolution transmission electron microscopy (HRTEM) images of PdNi/carbon nanofibers. Figure 5d shows typical STEM‐HAADF images of PdNi/carbon nanofibers. The figures show that the nanofibers have good morphological characteristics. Figure 5e,f illustrates that the electrodes display significant and rapid responses after adding glucose solutions. This phenomenon is observed for solutions with a constant concentration (0.2 mm) and with varying concentrations. Within 2 s, the electrodes achieve a steady‐state current of 95%. The experimental results show that the Pd‐Ni/CNF‐based nanofibers have improved redox performance and markedly increased electrocatalytic activity for sugar oxidation, which is more favorable than the other detectors reported thus far.

## 5 Characterization

It is important to characterize the metallized nanofibers first to ensure the successful synthesis of the morphology and structure. To identify nanofiber morphology, researchers typically employ SEM, TEM, HRTEM and high‐angle annular dark field‐scanning transmission electron microscopy (HAADF‐STEM) for nanofiber characterization.^[^ ^21^ ^]^ The images from this paper that are shown in a microscope exhibit the morphologies of nanofibers on one side and the uniform distribution of the metals modified on the nanofibers on the other side. Second, spectroscopic analysis, including Fourier‐transform infrared (FTIR) and UV–visible (UV–vis) spectroscopy, can determine the structural and group compositions of the synthesized metallized nanofibers. Third, the mechanical properties of the metallized nanofibers, including tensile strength, thermogravimetry analyses, Young's modulus, and elongation at break, should be characterized and determined. Finally, the diffraction pattern produced by the crystal can be measured by X‐ray diffraction experiments, reflecting the atomic distribution pattern inside the crystal. Of course, these characterization items are selected variously according to the different material compositions of metallized nanofibers and the function of the application.

## 6 Biomedical Applications

Metallic nanofiber‐based composites have unique nanomorphologies and metallic functionalities and they are uniquely suited for numerous biomedical applications. In this section, we summarize the applications of metallized nanofiber materials in biomedicine through the following five points. 1) Metallic nanofibers can be prepared in a three‐dimensional porous form with large specific surface areas and good adhesion properties. This structure is similar to the natural extracellular matrix morphology, and it can fill the cell‐tissue interstices to achieve a strong linkage. 2) The porous structure and strong adsorption properties of metallized nanofibers can deliver drugs. Metals can achieve in situ imaging and provide therapeutic effects, thus complementing drugs designed to be encapsulated or absorbed to obtain superior theranostic integration. 3) Metallized nanofiber composites are uniquely suited for the tumor treatments. Researchers can achieve tumor targeting, combination therapy, and sustained release of drugs based on a unique group design. 4) The application of biomaterials in the field of wound healing is a unique advantage. Nanofibers can be formed by encapsulating metals with antimicrobial activities or coating them with antioxidant natural materials, thus preventing wound infection and promoting wound repair. 5) The development of biosensors is another important area for metallized nanofibers. Biomedicine requires dynamic monitoring of the body in different conditions, so many metal‐based nanomaterials have been derived. These metallized nanofibers are stable, sensitive and well suited for the real‐time dynamic monitoring of H_2_O_2_, lactate, catechol, and sugar.

### 6.1 Tissue Engineering

**Figure 6:**
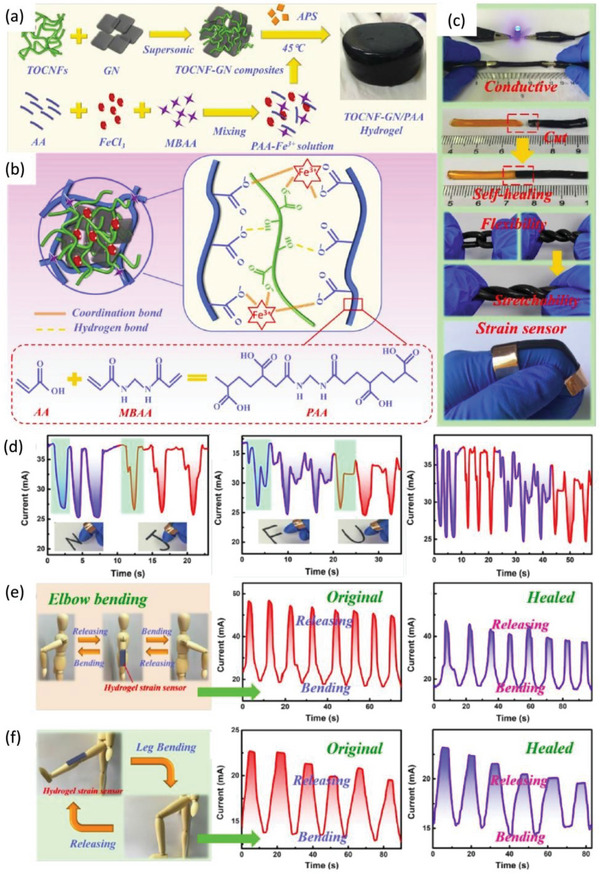
a) Schematic illustration of TOCNF‐GN/PAA hydrogels. b) Detailed preparation mechanism of TOCNF‐GN/PAA hydrogels. c) The results of characterization assays demonstrate that the TOCNF‐GN/PAA hydrogels has excellent electrical conductivity, self‐healing, flexibility, and stretchability. d) The strain sensor function of TOCNF‐GN/PAA hydrogel was utilized to change the current and therefore write different letters (N, J, F, and U) in the experiment. e) Application of the TOCNF‐GN/PAA hydrogel to the arms. f) Application of the TOCNF‐GN/PAA hydrogel to the legs. Reproduced with permission.^[^ ^24^ ^]^ Copyright 2020, Elsevier.

Tissue engineering and organ regeneration have received widespread attention, mainly in the fields of medicine, biology, and materials science. The human body is a relatively complex system that includes various important tissues and cellular matrices, and the system interacts with the whole human organism. Therefore, the preparation of biomaterials must be safe, stable, suitable for the complex microenvironment of the human body, and it must complement the corresponding tissues, organs, and body systems. Zheng et al. designed a polymerization method from basic chemistry that combines poly(3‐caprolactone) and 4‐dibenzocyclooctynol, thus enabling the design and synthesis of novel functionalizable nanobased scaffolds.^[^ ^22^ ^]^ Such functionalizable scaffolds can be used for various applications in the human body, and they have far‐reaching potential in the field of nanomedical biomaterials. Doench et al. designed and prepared nanofibers with nanoprofibrillated cellulose that can be used for viscous replenishment of nucleus pulposus.^[^ ^23^ ^]^ The design background of such nanofibers is based on the sol and gel states at the physical and chemical levels. This background suggests that the rheological behaviors related to the injectable sol state and the mechanically enhanced gel state of the fiber are included. In addition, in animal experiments, researchers have established pig and rabbit spine models and injected CNF/CHI formulations into the intervertebral disc to investigate the effects of the formulations on disc biomechanics. The experimental results show that with the injection of the preparation into the intervertebral disc to form a gel, the positioning is positioned at the injection site while restoring the disc viscoelasticity and restoring or increasing the disc height. The formulation support through the cell matrix can cushion a compressed nerve and provide therapeutic pain relief for patients. In addition, a TOCNF‐GN/PAA hydrogel with stretchable and conductive properties was prepared by Zheng et al. (**Figure** **6**a,b).^[^ ^24^ ^]^ By employing both physical and chemical double cross‐linking techniques, Fe^3+^ and N, N'‐methylenebis acrylamide were used to polymerize acrylic acid monomers in situ and create a dual‐crosslinked TOCNF‐GN/PAA composite hydrogel. This process enhances the mechanical properties of the hydrogel, and imbues it with an intrinsic ability to self‐heal. Figure 6c demonstrates that the TOCNF‐GN/PAA composite hydrogel has excellent electrical conductivity, self‐healing ability, flexibility, and stretchability. Figure 6d demonstrates that the strain sensor of the TOCNF‐GN/PAA hydrogel can change the current well; thus, the corresponding letter is written. The excellent reproducibility and sensitivity of the TOCNF‐GN/PAA hydrogel strain sensor self‐healing properties were confirmed through repetitive bending of both the original and self‐healed sensors attached to a human arm and leg. The strain sensor exhibits repetitive response behaviors, validating its self‐healing capabilities (Figure 6e,f). The hydrogel has an excellent self‐healing rate with a high mechanical strength, an elongation at break greater than 800%, and the ability to achieve a healing rate of more than 95% in half a day; thus, the hydrogel can be widely used as an ionic skin sensor in healthcare and wearable device applications. The above studies show that the three‐dimensional network structure of metallized nanofiber‐based materials is similar to that of human tissues, with good mechanical properties, biocompatibility, and regeneration ability, making it widely applicable in the fields of tissue engineering and wearable sensors.

### 6.2 Drug Delivery

**Figure 7:**
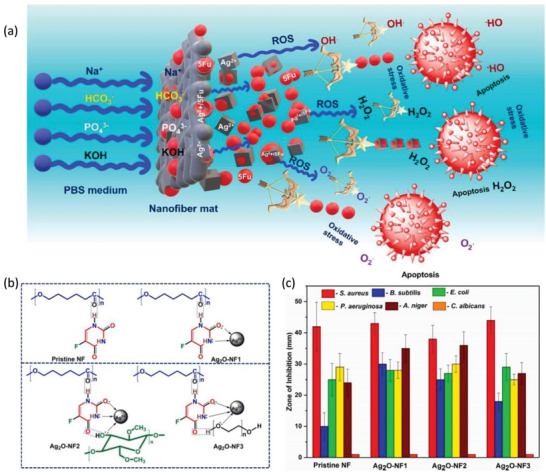
a) Schematic illustrates the mechanism by which a drug‐loaded nanofiber mat eliminates cancer cells by providing reactive oxygen species and inducing oxidative stress. b) Possible structural arrangements of nanofiber mats. c) Antimicrobial activity results of the nanofiber mats. Reproduced with permission.^[^ ^26^ ^]^ Copyright 2023, Elsevier.

Based on the porous structures of nanofibers, researchers have designed and developed various drugs for loading into delivery vehicles. By modifying the nanofibers, targeted drug delivery to the corresponding tissues and organs can be achieved. Implantable drug delivery systems, which is a class of controlled drug release systems that are surgically implanted or introduced subcutaneously, have received increasing attention and intensive research. Novel drug delivery modalities and their drug applications have expanded from reproductive health to various therapeutic areas, such as oncology treatment, ophthalmic diseases, insulin delivery, and cardiovascular diseases. In recent years, research interest in implantable drug delivery systems has continued to increase with advances in medical technology. Based on an implantable drug delivery system, Chen et al. prepared cisplatin composite micro/nanofibers and first focused on the release mechanism and profile of cisplatin.^[^ ^25^ ^]^ The experimental results show that the cisplatin‐based micro/nanofibers can well release cisplatin at the tumor site in a timely and quantitative manner, improving the retention time at the tumor site. Furthermore, the side effects of cisplatin, including renal and auditory complications, nausea, and vomiting, are reduced. This delivery system can be used for postoperative local chemotherapy and as an implantable drug for lung tumors in the future. Published in 2023, the authors prepare nanofiber mats containing nanoparticles that appeal to clinicians and provide excellent control over microbial infections while treating cancer (**Figure** **7**a).^[^ ^26^ ^]^ Nanoscale silver oxide with methylcellulose and polyethylene glycol are placed on polycaprolactone nanofiber pads by the electrospinning technique. The anticancer drug (5‐fluorouracil) loaded in the mat is released in a controlled manner for two weeks. Anticancer activity and apoptosis assays show that breast cancer cell lines are effectively eradicated by the release of ≈15% of the drug within 24 h and show profound cell viability against fibroblast cell lines. In addition, possible postsurgical microbial infections are strongly inhibited by the nanofiber pads. The fabricated nanofiber mats serve as promising candidates for drug delivery and tissue engineering implants. Figure 7b illustrates the possible structural arrangement of the nanofiber mats through various interactions. Figure 7c illustrates the antimicrobial properties of nanofiber, which is useful for the post‐surgical cancer therapy.

In addition to encapsulating drugs, nanofiber delivery systems can encapsulate small molecule contrast agents. Indocyanine green (ICG) has strong absorption in the near‐infrared region within 600 and 900 nm. The deep penetration of ICG is a very important advantage in imaging technology. However, when ICG dye is injected into the body through blood vessels, it binds to plasma proteins and is excreted from the body in a very short period. This feature becomes a disadvantage in targeted tissue imaging. Zeynep Rüya Eğe et al. encapsulated ICG dyes into biodegradable and biocompatible poly(ε‐caprolactone) polymers by a coaxial electrospinning method.^[^ ^27^ ^]^ The experimental results suggest that the nanofibers can improve the stability of the ICG dye in the biological environment and the residence time in the specific tissue. Therefore, this ICG dye‐based nanofiller can be used as an advanced nanoprobe for clinical medical imaging applications. Intelligent drug design, controlled drug release, and medical imaging delivery systems can address the full range of clinical medical requirements, including early detection and late‐stage treatment. Composite nanofiber materials can be prepared with sensitive photo‐responsiveness and pH‐responsiveness for controlled release drug properties; their sudden release effect at the early stage of drug retardation is significantly better than that of other similar materials. In addition, based on the matrix diversity levels of fiber materials, nanofibers can possess excellent biocompatibility and are suitable for biomedical applications. Therefore, nanofibers have many excellent properties and far‐reaching application prospects in drug delivery.

### 6.3 Tumor Treatment

In recent years, researchers have introduced many nanofibers for cancer therapy.^[^ ^28^ ^]^ Basically, nanofibers that encapsulate chemotherapeutic drugs in porous nanofibers use the properties of metals for thermal and magnetic therapy and are prepared as patches for surface tumor treatment. Arathyram Ramachandra Kurup Sasikala et al. exploited the material to prepare magnetic nanofibers (BTZ‐MMNF‐MH) as carriers for chemotherapy and thermotherapy against tumors.^[^ ^29^ ^]^ The device consists of monodisperse iron oxide nanoparticles (IONP) and the chemotherapeutic drug bortezomib, thus responding to the microenvironment. The IONP‐incorporated nanofiber matrix is prepared by an electrostatic spinning technique with poly(D,L‐lactide‐co‐glycolide). In addition, the bortezomib anticancer drug containing borates is bound by catechol metal in a pH‐sensitive manner. The high proliferation rates of normal cells on nanofibers indicate that nanofibers have excellent biocompatibility. The mussel‐inspired magnetic nanofiber has high cell proliferation relative to magnetic nanofiber and PLGA nanofiber. The employment of nanofibers attributed to polydopamine enhances the hydrophilicity of nanofibers to increase the adhesion of cells. In addition, in cellular experiments, synergistic antitumor treatment significantly increases apoptotic activity in the BTZ‐MMMF‐MH group, suggesting that this strategy is very promising for future clinical translational applications. The authors have successfully prepared implantable smart magnetic nanofibers that can combine thermal therapy and trigger drug release functions for tumor treatment. In clinical situations, smart nanofibers can be implanted near tumors by surgery, suggesting real‐time monitoring ability. Thus, these smart implantable nanofibers provide a safe route for the specific delivery of anticancer drugs to tumors and the retention of magnetic nanoparticles in the tumor environment for repetitive thermotherapy.

Wang et al. designed nanofibers for circulating tumor cells.^[^ ^30^ ^]^ Rather than encapsulating the drug, the authors design modifies the nanofibers with an epithelial cell adhesion molecule antibody to epithelial cell adhesion molecules; additionally, the design involves assembling electrode flexible electronic conduits composed of nanofibers and liquid metal‐polymer conductors, enabling nanofibers to specifically capture and kill peripheral cancer cells. The nanofibers established by the authors have many biomedical application scenarios to explore. The authors expect that they should try to establish a human‐derived tumor xenograft model in rabbits to investigate the roles of nanofibers in reducing the possibility of metastasis and recurrence. In addition to peripheral tumor cells, modifications of different antibodies may also enable nanofibers to function in the capture of different factors. Therefore, this study has wide application value.

**Figure 8:**
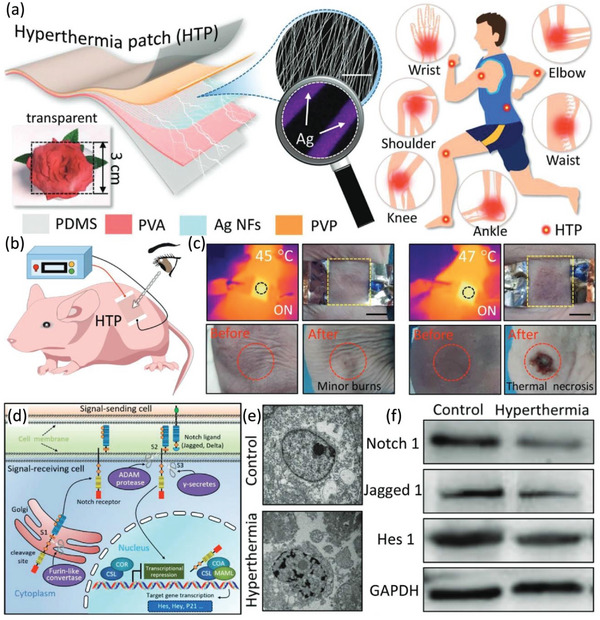
a) Soft broad‐spectrum transparent, homogeneous temperature distribution HTP for subcutaneous tumors. b,c) Schematic illustration of thermal therapy for subcutaneous tumors with HTP. Compare the efficacy of thermal therapy with HTP at two superheating temperatures of 45 and 47 °C. d) Schematic illustration of notch signaling pathways. e) TEM images demonstrating morphological changes in CAL‐27 cells. f) Western blotting assay showing changes in apoptosis‐related proteins. Reproduced with permission.^[^ ^31^ ^]^ Copyright 2022, John Wiley and Sons.

In addition, Wang et al. prepared a skin‐mountable hyperthermia patch (HTP) for the treatment of epidermal tumors.^[^ ^31^ ^]^ The thermotherapy patch consists of four main layers, which are the metalized Ag nanofiber network heating layer, PVA and PVP support layers, and the PDMS elastomer encapsulation layer (**Figure** **8**a). The authors established a subcutaneous tumor model in mice to determine the effect of the patch on tumors (Figure 8b). The authors compared the abnormal skin state at different temperatures (45 and 47 °C). The experimental results clearly show pale skin and thermal necrosis in the treated area (Figure 8c). In addition, the authors explored the potential mechanism of the patch leading to tumor apoptosis, exhibiting downregulated expression of Notch 1, Jagged 1, and Hes 1 proteins (Figure 8d–f). These results corroborate that HTP inhibits the growth of subcutaneous tumors by inducing cell apoptosis, and controlling the Notch signaling pathway. In this paper, the authors prepare patches for advanced tumors that may recur after radiation therapy. This device can solve the problem of tumor recurrence; additionally, it has many clinical applications as a novel special drug delivery option in certain areas, such as osteoarthritis, cervical spondylosis and chronic ocular surface inflammation.

In addition, based on the unique properties of nanofibers, nanofibers can be used as carriers for antitumor drug loading and to form short fibers by freeze‐cutting or homogenization, resulting in injectable nanofiber dispersions. It is believed that in the future, diverse nanofibers based on tumor treatment can be designed and synthesized into diverse forms, bringing advances to biomedicine.

### 6.4 Wound Healing

**Figure 9:**
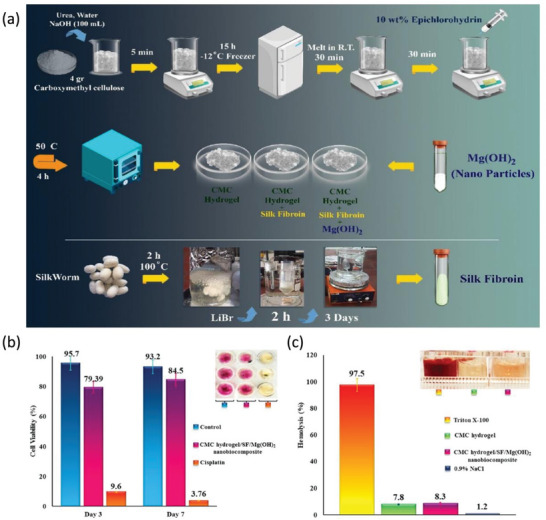
a) The synthesis process of CMC hydrogel/SF/Mg(OH)_2_ nanocomposite. b) Cell viability experiment with different treatment on the Hu02 cell line. c) Histogram and figure of cytolytic assay. Reproduced with permission.^[^ ^33^ ^]^ Copyright 2021, American Chemical Society.

Based on the antibacterial functions of metals and the multiple morphologies of nanofibers, researchers can prepare nanofibers with antibacterial effects for biomedical applications, such as wound healing.^[^ ^32^ ^]^ Reza Eivazzadeh‐Keihan et al. prepared a CMC hydrogel/SF/Mg(OH)_2_ nanocomposite scaffold with a hydrogel morphology that is cross‐linked with silk fibroin protein and antibacterial Mg(OH)_2_ nanoparticles (**Figure** **9**a).^[^ ^33^ ^]^ The authors selected the cellosaurus cell line Huo2 to investigate the biocompatibility of the nanocomplexes. The experimental results indicate that the cells cocultured with the nanocomplexes still show a cell survival rate reaching 84.5% after 7 days, indicating the nontoxic and harmless properties of the nanocomplexes (Figure 9b). Considering the hemolytic activity of the CMC hydrogel and the CMC/SF/Mg(OH)_2_ nanobiocomposite, no particular difference is observed (Figure 9c). In addition, the authors have determined the antibacterial activities of the nanocomplexes. The experimental results indicate that the nanocomplexes can completely limit the biofilm development of *Pseudomonas aeruginosa* on the surface, exhibiting excellent antibacterial activity. Nanocomposites can be considered novel and excellent candidates for research applications in biomedical fields such as tissue engineering. Another widely available antibacterial metal is silver. Silver is an effective antimicrobial agent because silver ions first create holes in the bacterial membrane and then enter the bacterium and bind core cellular components, such as DNA, thereby inhibiting the most basic biological functions of the bacteria.

By processing silver monomers or ions through nanotechnology, silver nanoparticles with particle sizes of 1–100 nm can be prepared. As a new inorganic antimicrobial material, Ag nanoparticles have excellent antimicrobial effects and safety levels that are significantly better than conventional antimicrobial agents. Ag nanoparticles have numerous antimicrobial activity, a long sterilization duration, and are nonresistant and safe. Furthermore, Ag nanoparticles have biological activities, such as antiviral and antitumor activities. To date, the preparation and application of Ag nanoparticles have become a popular topic for research on antibacterial materials. Karina Santiago‐Castillo et al. dispersed Ag nanoparticles in a chitosan matrix and immobilized their cubic structure after electrostatic spinning.^[^ ^34^ ^]^ The PVA‐CTS‐Ag nanoparticles prepared by the two‐step method have been experimentally demonstrated to be developed for antimicrobial and wound healing applications. In addition, some researchers have prepared Ag as Ag‐MOF, which can be loaded with some antibacterial and hemostatic drugs while stabilizing the structure. Chen et al. prepared a 3D‐layered nanofiber based on MOFs (3D‐AgMOF‐CUR) loaded with curcumin, promoting the wound exudate absorption, accelerated hemostasis, resisted bacterial growth, and inhibited inflammation.^[^ ^35^ ^]^ The authors established an in vivo wound infection model. The results indicate that 3D‐AgMOF‐CUR has superior antibacterial capabilities, dramatically promotes collagen deposition and tissue regeneration, accelerates wound healing, and has the potential to be an attractive alternative to conventional wound healing in future clinical applications. In addition, Pratibha Bhadauriya et al. synthesized carbon nanofibers dispersed with yeast extract and copper nanoparticles as a potential diabetic wound healing material (Cu‐CNF‐YE).^[^ ^36^ ^]^ Yeast has a role in promoting the function of the immune system and intestinal health of animals. In addition, the ethanol produced in the yeast fermentation process has disinfection and sterilization effects, with favorable application prospects. The authors demonstrate that the nontoxic Cu‐CNF‐YE has excellent antibacterial effects and can greatly improve wound healing in patients with diabetes. The innovation of the authors provides guidance for prospective applications of nanofibers in wound healing. Metal ion‐incorporated nanofibers for tissue regeneration, have many advantages. For example, Zn^2+^ ions can promote cell proliferation and differentiation, contributing to tissue regeneration and repair. In addition, Zn^2+^ ions have antibacterial properties, preventing infection and promoting wound healing. Mg^2+^ ions can promote the proliferation and differentiation of bone cells and contribute to bone tissue regeneration. In addition, Mg^2+^ ions can promote the repair and regeneration of soft tissues. Cu^2+^ ions can promote angiogenesis and cell proliferation, thus regenerating and repairing tissues. In addition, Cu^2+^ ions have antibacterial properties that can prevent infection. These metal ion‐conjugated nanofibers can modulate therapeutic effects by controlling the rate and amount of release, thus promoting tissue regeneration and repair. Furthermore, they have good biocompatibility and do not cause immune or rejection reactions. Overall, metal ion‐incorporated nanofibers have great potential in tissue regeneration and can be used in various applications, such as wound healing, bone regeneration, and tissue engineering.

### 6.5 Biosensors

Nanofibers possess smaller diameters, larger specific surface areas, and longer lengths than other metallic nanomaterials, which are suitable for applications in the fields of sensors. Biosensors generally consist mainly of a biosensitive element and a transducer, of which the biosensitive element is the most essential part. The advantage of utilizing high‐voltage electrostatic spinning as a biosensitive element is that the spinning material has excellent biocompatibility and can be used in various manners to self‐assemble spinning fibers with biological macromolecules and to achieve specific recognition of the measured object. Manesh et al. immobilized glucose oxidase on PMMA electrospun composite films dispersed with carbon nanotubes.^[^ ^37^ ^]^ The carbon nanotubes are functionally modified by PDDA to carry a positive charge. Through electrostatic interactions, the negatively charged glucose oxidase is immobilized on the carbon nanotubes, resulting in the efficient electron transfer and improved reproducibility of the assay and stability of the complex electrodes. To obtain chemical sensors with high sensitivity and excellent performance, materials that can specifically interact with molecules in the gas or solution and produce changes in electrical or optical properties are generally selected. It usually includes the following two methods: 1) obtaining polymer blends of semiconductor oxides by electrospinning and then removing the polymer to obtain nanowires or nanoribbons of semiconductor oxides and 2) sputtering a layer of semiconductor metal oxides on the outer layer of polymer electrospinning, and then calcining at high temperature to remove the polymer, which can conveniently obtain hollow semiconductor fibers with a large length‐to‐diameter ratio. **Table** **1** summarizes the applications of biosensors. With the continued maturation and development of electrostatic spinning technology, nanofibers with excellent structures are ideal for biosensors. In the future, there is an increasing number of electrostatic nanofiber sensors with high sensitivity and practical application value that are developed to contribute to the biomedical field and human life.

**Table 1:** Metallized nanofibers strategies for biosensor properties

Metals/metal oxide/alloy	Nanofibers	Applications	Reference
Pd	Pd‐helical carbon nanofibers	Biosensor for H_2_O_2_ and glucose by means of cyclic voltammetry and chronoamperometry	[38]
Pd	Pd nanoparticles‐decorated carbon nanofibers	Biosensor for detection of H_2_O_2_ and nicotinamide adenine dinucleotide	[39]
Pt	Pt nanoparticles‐carbon nanofibers	Biosensor applied to the quantification of lactate in real sweat samples	[19]
Ni	Polydopamine‐laccase‐nickel‐nanoparticle modified with carbon nanofibers	For biosensing of catechol	[21]
Rh	Rh nanoparticles‐decorated carbon nanofibers	Biosensor for detecting hydrazine content	[40]
Cu	Cu nanoparticles‐loaded carbon nanofibers composite	For the detection of catechol	[41]
ZnO/SnO_2_	ZnO/SnO_2_ nanonodules‐decorated carbon nanofibers	For dimethyl methylphosphonate gas detection	[42]
WO_3_	WO_3_ nanonodule decorated carbon nanofibers	For NO_2_ gas sensor application	[43]
Pd‐Ni alloy	Pd‐Ni alloy nanoparticles/carbon nanofibers composite	For sugar content detection in flow systems	[20]
MCo (M = Fe, Cu, Mn, and Ni) alloy	MCo nanoparticles‐decorated carbon nanofibers	For glucose detection in human serum samples	[44]

## 7 Conclusions

Metallized nanofibers are rapidly being developed.^[^ ^45^ ^]^ From the perspective of the chemical field, metallized nanofibers can store energy for development into solar cells. In addition, metallized nanofibers can be functionalized by various materials and thus applied to many catalytic and biomimetic fields.^[^ ^46^ ^]^ In this review, the preparation concepts and material applications of metallized nanofibers in the biomedical field are highlighted. First, we summarize the advantages of metals in nanofibers, including strong mechanical properties, providing a foundation for the subsequent construction of drug‐loaded imaging, medical devices, etc. Then, we introduce the preparation and modification of nanofibers based on biomedical applications, which require materials that are nontoxic, harmless, and bacteria‐free. Next, the morphological classification of metallized nanofibers and their basic characterization are discussed. Finally, the review highlights the biomedical applications of metallized nanofibers. As observed in many studies on metallized nanofibers, there are numerous developments in response to the needs of researchers, including metal‐organic framework‐based, metal‐phenolic networks‐based, hydrogel‐based, and nanoparticle‐functionalized nanofibers. Each of these metallized nanofibers has unique characteristics and active applications in nanopharmaceutical delivery, tumor therapy, wound repair, and biosensing. In addition, metallized nanofibers can combine the properties of various metallic materials, such as the thermal therapy of ferromagnetic materials, the multicombination treatment mode of nanogold, the antibacterial capability of metallic silver nanoparticles, and the flexibility to adapt to various different drugs and targeted peptides; these fibers can be extended to the development of various nanomedicines with different specificities and the treatment of different types of diseases. Finally, research on nanofibers is better developed in the detection section regarding the encapsulated drug efficiency, and the release process. Researchers still need to explore improved detection mechanisms for this section. Metallized nanofibers can be used to prepare various biomedical materials, such as artificial heart valves, artificial blood vessels, and tissue engineering scaffolds. In addition, metal nanofibers can be used as biosensors and drug carriers, which are important for biomedical research and treatment.

Metallized nanofibers are promising as materials with excellent properties in the biomedical field, and there are still some limitations. Therefore, future improvement in the preparation and application of metallized nanofibers can be considered from the following aspects. 1) The safety of metallic nanofibers cannot be ignored. Because of the biomedical applications concerned, the materials selected by the researchers must be nontoxic and nonhazardous, with particular attention to the side effects of the metal, the metabolic modalities, and the possible bodily immune response. 2) Further optimization of the synthesis method of metallized nanofibers and the modification of multiple sites is necessary. Nanofibers can be modified by specific functional groups (carboxyl, amino, amide, alkoxy, sulfonic acid groups, etc.) during surface modification to broaden the applications of metallized nanofibers. In addition, with the development of artificial intelligence, the preparation process can be combined with artificial intelligence for optimal selection. 3) The design of metallized nanofiber materials can be enhanced in various fields, including materials science, nanotechnology, pharmacy, and clinical medicine. Researchers need to expand their application field and focus on solving practical issues in the pharmaceutical industry. 4) Clinical translation has always been a challenge for nanomaterials. A method for producing metallized nanofibers industrially while ensuring that their products are stable, safe, and controllable is a major issue for researchers to overcome. Despite these drawbacks, metallized nanofibers still have promising applications as technology advances and the demand for applications increases.

## Conflict of Interest

The authors declare no conflict of interest.

## References

[1] M. Furutani , E. Fujii , K. Ogura , Mater. Trans. 2021, 62, 1457.

[2] K. Mondal , M. A. Ali , V. V. Agrawal , B. D. Malhotra , A. Sharma , ACS Appl. Mater. Interfaces 2014, 6, 2516. 24447123 10.1021/am404931f

[3] R. Xue , M. T. Nelson , S. A. Teixeira , M. S. Viapiano , J. J. Lannutti , Biomaterials 2016, 76, 208. 26524540 10.1016/j.biomaterials.2015.10.055

[4] A. Theron , E. Zussman , A. L. Yarin , Nanotechnology 2001, 12, 384.

[5] Kenry , C. T. Lim , Prog. Polym. Sci. 2017, 70, 1.

[6] a) J. Heo , G. Lim , J. Sens. Sci. Technol. 2015, 24, 35;

[7] a) S. Wang , C. Wang , B. Zhang , Z. Sun , Z. Li , X. Jiang , X. Bai , Mater. Lett. 2010, 64, 9;

[8] a) J. Chen , Z. Li , D. Chao , W. Zhang , C. Wang , Mater. Lett. 2008, 62, 692;

[9] a) W. Zhang , G. Cai , R. Wu , Z. He , H. B. Yao , H. L. Jiang , S. H. Yu , Small 2021, 17, 2004140; 10.1002/smll.202004140 33522114

[10] W. Li , Y. Li , X. Wen , Y. Teng , J. Wang , T. Yang , X. Li , L. Li , C. Wang , J. Membr. Sci. 2022, 648, 120369.

[11] W. Zhang , Z. Y. Wu , H. L. Jiang , S. H. Yu , J. Am. Chem. Soc. 2014, 136, 14385. 25244060 10.1021/ja5084128

[12] B. Dey , M. W. Ahmad , G. Sarkhel , D. J. Yang , A. Choudhury , Mater. Sci. Semicond. Process. 2022, 142, 106500.

[13] Z. Zhang , L. Xie , Y. Ju , Y. Dai , Small 2021, 17, 2100314. 10.1002/smll.202100314 34018690

[14] Q. Li , W. Xiao , F. Zhang , Q. Liu , J. Ye , H. Dong , X. Cao , J. Mater. Chem. B 2018, 6, 2734. 32254226 10.1039/c8tb00350e

[15] T. Ghosh , T. Das , R. Purwar , Polym. Eng. Sci. 2021, 61, 1887.

[16] M. Mohammed , R. D. Chakravarthy , H.‐C. Lin , Mol. Syst. Des. Eng. 2022, 7, 1336.

[17] Y. Y. Li Sip , D. W. Fox , L. R. Shultz , M. Davy , H.‐S. Chung , D.‐X. Antony , Y. Jung , T. Jurca , L. Zhai , ACS Appl. Nano Mater. 2021, 4, 6045.

[18] S. S. Muthu , Polymer Technology in Dye‐containing Wastewater, Springer, Hong Kong 2022.

[19] P. J. Lamas‐Ardisana , O. A. Loaiza , L. Anorga , E. Jubete , M. Borghei , V. Ruiz , E. Ochoteco , G. Cabanero , H. J. Grande , Biosens. Bioelectron. 2014, 56, 345. 24534552 10.1016/j.bios.2014.01.047

[20] Q. Guo , D. Liu , X. Zhang , L. Li , H. Hou , O. Niwa , T. You , Anal. Chem. 2014, 86, 5898. 24837693 10.1021/ac500811j

[21] D. Li , L. Luo , Z. Pang , L. Ding , Q. Wang , H. Ke , F. Huang , Q. Wei , ACS Appl. Mater. Interfaces 2014, 6, 5144. 24606719 10.1021/am500375n

[22] J. Zheng , S. Xie , F. Lin , G. Hua , T. Yu , D. H. Reneker , M. L. Becker , Polym. Chem. 2013, 4, 2215.

[23] I. Doench , M. E. W. Torres‐Ramos , A. Montembault , P. Nunes de Oliveira , C. Halimi , E. Viguier , L. Heux , R. Siadous , R. Thire , A. Osorio‐Madrazo , Polymers 2018, 10, 1202. 30961127 10.3390/polym10111202 PMC6290636

[24] C. Zheng , K. Lu , Y. Lu , S. Zhu , Y. Yue , X. Xu , C. Mei , H. Xiao , Q. Wu , J. Han , Carbohydr. Polym. 2020, 250, 116905. 33049881 10.1016/j.carbpol.2020.116905

[25] P. Chen , Q. S. Wu , Y. P. Ding , Z. C. Zhu , Nano 2011, 6, 325.

[26] E. Jaisankar , R. S. Azarudeen , M. Thirumarimurugan , J. Drug Delivery Sci. Technol. 2023, 84, 104451.

[27] Z. R. Ege , A. Akan , F. N. Oktar , C. C. Lin , D. S. Kuruca , B. Karademir , Y. M. Sahin , G. Erdemir , O. Gunduz , J. Biomed. Mater. Res., Part B 2020, 108, 538. 10.1002/jbm.b.34410 31087780

[28] a) L. Chen , X. Sun , K. Cheng , P. D. Topham , M. Xu , Y. Jia , D. Dong , S. Wang , Y. Liu , L. Wang , Q. Yu , Adv. Fiber Mater. 2022, 4, 1669;

[29] A. R. K. Sasikala , A. R. Unnithan , Y. H. Yun , C. H. Park , C. S. Kim , Acta Biomater. 2016, 31, 122. 26687978 10.1016/j.actbio.2015.12.015

[30] D. Wang , R. Dong , X. Wang , X. Jiang , ACS Nano 2022, 16, 5274. 35302351 10.1021/acsnano.1c09807

[31] Q. Wang , H. Sheng , Y. Lv , J. Liang , Y. Liu , N. Li , E. Xie , Q. Su , F. Ershad , W. Lan , J. Wang , C. Yu , Adv. Funct. Mater. 2022, 32, 2111228.

[32] a) P. Yudaev , Y. Mezhuev , E. Chistyakov , Gels 2022, 8, 329; 35735673 10.3390/gels8060329 PMC9222824

[33] R. Eivazzadeh‐Keihan , F. Khalili , N. Khosropour , H. A. M. Aliabadi , F. Radinekiyan , S. Sukhtezari , A. Maleki , H. Madanchi , M. R. Hamblin , M. Mahdavi , S. M. A. Haramshahi , A. E. Shalan , S. Lanceros‐Mendez , ACS Appl. Mater. Interfaces 2021, 13, 33840. 34278788 10.1021/acsami.1c07285

[34] K. Santiago Castillo , A. M. Torres Huerta , D. Del Angel Lopez , M. A. Dominguez Crespo , H. Dorantes Rosales , D. Palma Ramirez , H. Willcock , Polymers 2022, 14, 674. 35215587 10.3390/polym14040674 PMC8880230

[35] J. Chen , Z. Huang , H. Zhang , Z. Zhang , D. Wang , D. Xia , C. Yang , M. Dong , Chem. Eng. J. 2022, 443, 136234.

[36] P. Bhadauriya , H. Mamtani , M. Ashfaq , A. Raghav , A. K. Teotia , A. Kumar , N. Verma , ACS Appl. Bio Mater. 2018, 1, 246. 10.1021/acsabm.8b00018 35016382

[37] K. M. Manesh , H. T. Kim , P. Santhosh , A. I. Gopalan , K. P. Lee , Biosens. Bioelectron. 2008, 23, 771. 17905578 10.1016/j.bios.2007.08.016

[38] X. Jia , G. Hu , F. Nitze , H. R. Barzegar , T. Sharifi , C. W. Tai , T. Wagberg , ACS Appl. Mater. Interfaces 2013, 5, 12017. 24180258 10.1021/am4037383

[39] J. Huang , D. Wang , H. Hou , T. You , Adv. Funct. Mater. 2008, 18, 441.

[40] G. Hu , Z. Zhou , Y. Guo , H. Hou , S. Shao , Electrochem. Commun. 2010, 12, 422.

[41] J. Fu , H. Qiao , D. Li , L. Luo , K. Chen , Q. Wei , Sensors 2014, 14, 3543. 24561403 10.3390/s140203543 PMC3958252

[42] J. S. Lee , O. S. Kwon , S. J. Park , E. Y. Park , Y. Sun Ah , ACS Nano 2011, 5, 7992. 21905727 10.1021/nn202471f

[43] J. S. Lee , O. S. Kwon , D. H. Shin , J. Jang , J. Mater. Chem. A 2013, 1, 9099.

[44] M. Li , L. Liu , Y. Xiong , X. Liu , A. Nsabimana , X. Bo , L. Guo , Sens. Actuators, B 2015, 207, 614.

[45] L. Li , R. Hao , J. Qin , J. Song , X. Chen , F. Rao , J. Zhai , Y. Zhao , L. Zhang , J. Xue , Adv. Fiber Mater. 2022, 4, 1375.

[46] a) W. Huang , Y. Xiao , X. Shi , Adv. Fiber Mater. 2019, 1, 32;

